# Making the Model

**Published:** 1906-06

**Authors:** Stewart J. Spence

**Affiliations:** Chattanooga, Tenn.


### MAKING THE MODEL.

By Stewart J. Spence, Chattanooga, Tenn.

Having taken the impression, as described in the first paper of this series, the next step is to prepare it for the model.

If the impression is of plaster and has fractured, its parts should be freed of all little chips by being held under running water, then carefully adjusted to their original positions. After this separate them again and touch Sandarac varnish to the fractures, then reassemble them. It is well to reinforce this by melted wax poured over the fracture. As wax contracts largely in cooling down from the melting point, it sometimes becomes necessary to maintain the parts in position by the fingers till the wax is cold. In extreme cases, invest around the fractured parts with plaster, instead of wax.

The lingual space of the lower impression should be filled with plaster, before pouring model, in order that the model may be the stronger, because thus it will bridge said space instead of being horse-shoe shaped.

Cut down impression along buccal, labial and lingual borders till these are no higher than the plate shall be.

Examine the patient's jaws, and if either the palate or ridge show small hard places, deftly shave the impression at these points. It often happens that ridges are thus irregularly hard. If the hard parts are extensive, say, reaching over more than an inch of surface, and especially if they extend to the posterior border of plate, do not attempt the shaving. If the upper jaw is hard along the suture, use for relief chamber a strip of thin tin plate about half an inch wide and as long as said hard part, and extending back to within a quarter inch of posterior border of plate, which in such case should reach well on toward the soft palate, in order that said quarter inch of plate may not be made to rest on the hard portion. Some well-known authorities advocate the carrying of the border of plate far onto the soft palate, carving model so that said border will impinge the soft tissues there. The writer cannot speak on this method from experience, not having tried it because not having found it necessary.

*For separating medium* a solution of soap is perhaps best, as it yields the smoothest and (if left to thoroughly dry before pouring model) the hardest surface of model. This is because a scum of it remains on the surface of the impression, while oil sinks in. If impression has lain for a day or so, better soak it in water before applying separating medium. A very dry impression, by absorbing water from the plaster poured to make model, prevents easy flow of said plaster, and it is apt to be difficult to separate from model. On the other hand, too much moisture on the impression, whether from varnish, oil, soap or water, is taken up by the model and gives it a soft surface to slight depth. Energetic rapping of tray on bench, while pouring model, increases surface hardness of model considerably, because it shakes upward from the bottom many of the air bubbles which inhabit all plaster casts. When such rapping is contemplated, to prevent overrun of plaster, pin strip of paper around impression.

To pour the model, mix plaster of Paris (if this material is used) with as little stirring as possible, because long stirring increases expansion, and of medium consistency, because this gives greatest hardness. To avoid air holes, place thimbleful of this mix in center of impression and make it spread out by rapping tray on bench. To insure the filling of tooth holes, punch plaster into them with tapering instrument, otherwise imprisoned air may cause defective model teeth.

As all plaster of Paris expands in setting (usually about one per cent of its diameter) if it has space in which to expand, and as if this expansion is restricted or altogether prevented from extending laterally (lay the flanges of the tray in the case of the impression and lay the buccal surfaces of the impression in the case of the model) it then finds expression in a bowing up of the palatal arch; therefore the dentist should, before separating model from impression, trim their posterior borders, and that of the impression down to the tray, to determine if any considerable space exists between tray and impression, or impression and model. If so, better discard them; as failure is assured; and try again. Better still use Spence's Plaster, which is non-expanding. Sometimes this bowing up of the plaster of Paris casts is not very bad, as though the molecules had been compressed more closely together by a resistance to expansion, this arising either from the buccal surfaces, as aforesaid, or the thickness of plaster over palate.

To facilitate separation of plaster impression and model, place them in boiling water for a few minutes. If teeth remain, the impression must be carefully cut away. To indicate when the model is being approached in so doing, the impression ought, before pouring model, to have been painted with ink of thin shellac varnish at points of danger. If the impression be of Modeling Compound it had better be lightly soaped or oiled before pouring model, else, if overheated, little films of it will stick tenaciously to model. When this occurs, to remove said films, apply to them some modeling compound quite hot, holding it there till cooled, which may be done in water; when it will have adhered to the film on the model and come away with it.

If the impression has been in modeling compound, its slight contraction will tend to cause the plate to hug the buccal surfaces tighter, but if in plaster, which often expands but never contracts in setting, it is well to paint with a coat or two of varnish the buccal and labial walls of the impression (if upper), as this increases slightly the adhesion of even an exactly fitting plate.

The harder the mouth, if the plate fits it exactly, the stronger the adhesion; as is illustrated by grinding two plates of glass together; for the more they are ground to exact contact, the less can air penetrate, and therefore the greater the "suction." Therefore a non-expanding plaster suits them best. But most mouths are not very hard, and are frequently soft on the dental ridge, which is the place where pressure falls, and for these the contraction of wax or modelling compound, or the coating the ridge (as well as the before mentioned coating of the buccal and labial surface) with varnish, is advisable.

If impression and model are of expanding plaster, said coating is especially desirable, and often the throwing a bead around the entire border of the plate is of advantage. Said bead, however, will not be tolerated by the membrane when the fit is exact.

